# Essential Oils from Apiaceae, Asteraceae, Cupressaceae and Lamiaceae Families Grown in Serbia: Comparative Chemical Profiling with In Vitro Antioxidant Activity

**DOI:** 10.3390/plants12040745

**Published:** 2023-02-07

**Authors:** Nevena Gladikostić, Bojana Ikonić, Nemanja Teslić, Zoran Zeković, Danica Božović, Predrag Putnik, Danijela Bursać Kovačević, Branimir Pavlić

**Affiliations:** 1 Faculty of Technology, University of Novi Sad, Blvd. Cara Lazara 1, 21000 Novi Sad, Serbia; 2 Institute of Food Technology, University of Novi Sad, Blvd. Cara Lazara 1, 21000 Novi Sad, Serbia; 3 Department of Food Technology, University North, Trg dr. Žarka Dolinara 1, 48000 Koprivnica, Croatia; 4 Faculty of Food Technology and Biotechnology, University of Zagreb, Pierottijeva 6, 10000 Zagreb, Croatia

**Keywords:** medicinal plant, essential oil, hydrodistillation, antioxidant activity, principal component analysis (PCA)

## Abstract

The aim of the present study was to investigate the chemical profile and antioxidant activity of essential oils obtained from the most commonly grown plant species in Serbia. Aromatic and medicinal plants from Lamiaceae (*Mentha x Piperita*, *Ocimum basilicum*, *Origanum majorana*, *Origanum vulgare*, *Salvia officinalis*, *Satureja hortensis*, *Satureja montana* and *Thymus vulgaris*), Asteraceae (*Ehinacea purpurea* and *Matricaria chamomilla*), Apiaceae (*Anethum graveolens*, *Carum carvi*, *Foeniculum vulgare*, *Petroselinum crispum* and *Pimpinella anisum*) and Cupressaceae (*Juniperus comunis*) were selected as raw material for essential oils (EOs)’ isolation. Hydrodistillation (HD) was used for the isolation of EOs while they were evaluated in terms of yield and terpenoid profiles by GC-MS. In vitro radical scavenging DPPH and ABTS^+^ radical activities were carried out for all EOs. Finally, a principal component analysis (PCA) was performed with the experimental results of the composition and antioxidant activity of the EOs, which showed a clear distinction between the selected plant species for the aforementioned responses. This work represents a screening tool for the selection of other EO candidates for further processing by emerging extraction techniques and the use of EOs as natural additives for meat products.

## 1. Introduction

The Republic of Serbia has rich unexplored herbal biomes with plants that are excellent raw material for the production of essential oils (EOs) and for other valuable biologically active compounds [1]. Serbia has an extreme continental climate with dry air, where plants belonging to the various families (peppermint, sage, thyme, basil, chamomile, fennel, caraway, juniper, etc.) are usually cultivated or collected in the wild.

The EOs, as a source of natural products, represent a promising substitute for synthetic active ingredients in the food, pharmaceutical, cosmetic, alternative medicine and aromatherapy industries [2]. They are commonly associated with various physiological activities, including antibacterial, antiviral, anti-inflammatory, antifungal, antimutagenic, anticarcinogenic and various antioxidant activities [3]. EOs are volatile liquids obtained from different plant parts, including flowers, roots, bark, leaves, seeds, peels, fruits, wood and herbs [3]. Usually, they consist of lipophilic and highly volatile secondary plant compounds. In general, the main constituents of EOs consist of mono- and sesqui-terpenes, which are usually in hydrocarbon or oxygenated form. The uses of EOs are highly diverse and depend on various factors such as source, quality, extraction procedure, etc. Most frequently, they are used in the production of perfumes, cosmetics, soap, shampoos or cleansing gels [4]. Another interesting aspect of these oils is their potential as therapeutic agents in aromatherapy or as active ingredients or excipients in pharmaceuticals [4]. Another important application of EOs is the agri-food industry, both for the production of beverages and for the flavoring of foods [5]. Additionally, EOs and terpenoid-rich extracts from aromatic and medicinal plants have been increasingly recognized as promising natural agents for partial or complete replacements for synthetic and conventional preservatives in meat products [6].

The Lamiaceae or mint family, that is widely spread in natural ecosystems, consists of 6000 species in 236 genera. These aromatic plants have square stems in the cross-section, opposite leaves, zygomorphic flowers with five united petals and sepals and are cultivated for their ease of propagation, i.e., by cutting the stems. The most common genera include *Lavandula* L., *Melissa* L., *Mentha* L., *Ocimum* L., *Origanum* L., *Perilla* L., *Rosmarinus* L., *Salvia* L., *Satureja* L. and *Thymus* L. [7]. Many species from the Lamiaceae family possess high-quality EOs found in all aerial parts, especially in leaves and flowers. They are characterized by effective antibacterial, antifungal, antioxidant, antiviral and even anticancer properties and they are commonly used in the medicinal, cosmetic and perfume industries [8].

The Asteraceae family includes a large number of flowering plants grouped into nearly 1600 genera with over 23,000 species. Some of the most commonly used species are chamomile (*Matricaria recutita* L.), echinacea (*Echinacea purpurea* L.), yarrow (*Achillea millefolium* L.) and wormwood (*Artemisia absinthium* L.) [9]. The most striking and remarkable feature of the Asteraceae genera is that their flowers are characteristically grouped in compact inflorescences (heads) [10]. Plants from this family have EOs that are mostly found in the flowers and colored lightly green to darkish blue hues (due to chamazulene formed during distillation) [11]. The EOs and extracts of these species exhibit antioxidant, antibacterial, antifungal, antimicrobial and herbicidal activities [11].

The Apiaceae family is one of the most important families of flowering plants, consisting of 3780 species in 434 genera. The most commonly cultivated plants are cumin (*Cuminum cyminum* L.), caraway (*Carum carvi* L.), fennel (*Foeniculum vulgare* Mill.), coriander (*Coriandrum sativum* L.), anise (*Pimpinella anisum* L.), dill (*Anethum graveolens* L.) and parsley (*Petroselinum crispum* L.) [12]. Typically, EOs can be extracted from all parts of Apiaceae plants, but most commonly the greatest amounts are found in their seeds. Their oils are yellowish to colorless with a fresh fragrance. Regularly, their EOs are used for food flavors, in perfume and dyeing industries or for the manufacturing of soaps and detergents. They have important biological activities such as antitumor, antimicrobial, anti-inflammatory, analgesic, free radical scavenging, diuretic, gastrointestinal and anti-obesity properties [13].

The Cupressaceae family includes 27–30 genera with a total of about 130–140 species. They are monoecious, subdioecious or (rarely) dioecious trees and shrubs up to 116 m tall. Junipers are among the most important evergreen shrubs, ground covers and small evergreen trees. The berries contain EOs with a characteristic conifer-like aroma and bitter taste. The EOs from these species possess diuretic, antiseptic, carminative, anthelmintic, antibacterial, antifungal, antiviral, antioxidant, anti-inflammatory, antirheumatic and other properties [14].

Hydrodistillation (HD) is the simplest and oldest method for isolating EOs [15]. To that end, Clevenger’s system HD is recommended in the 3rd edition of the *European Pharmacopoeia* for the determination of the contents of EOs [16]. Even though HD has disadvantages in terms of longer extraction times, and lower yields needing larger amounts of raw herbal material, etc. [17,18], when HD is compared to the advanced extraction techniques (microwave assisted, supercritical, etc.), it is still the most common industrial isolation approach for EOs due to its simple facilities (no expensive equipment required), ease of implementation and selectivity. Hence, it still holds great importance for industrial applications.

Because of the aforesaid reasons, the present study was intended to broaden the limited existing data regarding the chemical composition and antioxidant activity of several EOs that should be helpful for industrial production while employing conventional HD. Hence, this research targeted the isolation and profiling of the pure EOs of Apiaceae, Asteraceae, Cupressaceae and Lamiaceae species grown in Serbia in order to report the influences of the different plant families on the total extraction yields, chemical compositions, bioactivity and antioxidant potential of the extracted EOs. A principal component analysis (PCA) was utilized to correlate these variables with EO samples. The aim of this was to provide a platform for the selection of EO candidates which could be applied as natural additives in various food products, particularly meat products, and nutraceuticals.

## 2. Results and Discussion

### 2.1. EO Yield

To ensure the intensification of mass transfer, the plant material should be crushed to an appropriate particle size, which is usually in the range of 100 µm–2 mm [19]. It is known that with a larger contact area, extraction efficiency increases. Moreover, finer particles improve the mass transfer rate from the solid to the liquid phase [20]. It is recommended that the diameter of the plant material is not larger than 2 mm and smaller than 0.5 mm (<10%). The particle size of all samples is shown in Table 1. The plant material was properly prepared, and it contained only 4.35% of particles above the established upper limit (2 mm). The fraction of fine particles was less than 0.5 mm in diameter and accounted for 8.69% of the sample. Finally, the mean particle size of the processed samples was 1.07 mm. The moisture contents of the samples are presented in Table 1. In many cases, the extraction yield improves by the moisture of the matrix, which acts as a solvent. The moisture in the plant material heats, evaporates and increases the internal pressure, causing the cell rupture and release of the solutes and resulting in a higher extraction yield [20].

**Figure 1:**
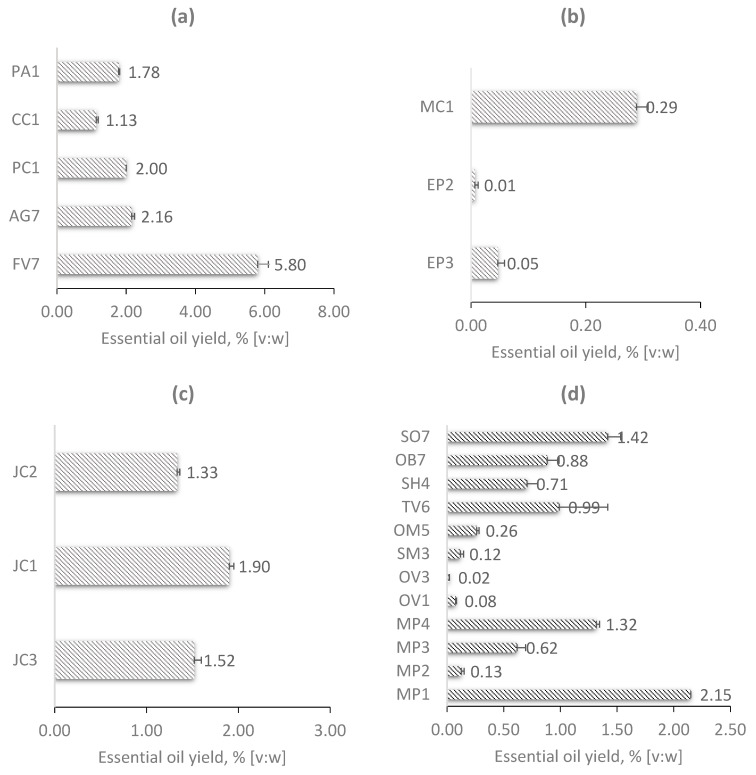
EOs’ yield: (**a**) Apiaceae species, (**b**) Asteraceae species, (**c**) Cupressaceae species, (**d**) Lamiaceae species; PA1—*Pimpinella anisum* from Institute for Medicinal Plant Research “Dr Josif Pancic”; FV7—*Foeniculum vulgare* from agricultural holding, Bačko Novo Selo; CC1—*Carum carvi* from Institute from Medicinal Plant Research “Dr Josif Pancic”; AG7—*Anethum graveolens* from agricultural holding, Bačko Novo Selo; PC1—*Petroselinum crispum* from Medicinal Plant Research “Dr Josif Pancic”; MC1—*Matricaria chamomilla* from Institute for Medicinal Plant Research “Dr Josif Pancic”; EP2—*Ehinacea purpurea* from Adonis d.o.o.; EP3—*Ehinacea purpurea* from Bilje Borča d.o.o.; JC2—*Juniperus comunis* from Adonis d.o.o.; JC3—*Juniperus comunis* from Bilje Borča d.o.o.; JC1—*Juniperus comunis* from Institute for Medicinal Plant Research “Dr Josif Pancic”; MP1—*Mentha piperita* from Institute for Medicinal Plant Research “Dr Josif Pancic”; MP2—*Mentha piperita* from Adonis d.o.o.; MP3—*Mentha piperita* from Bilje Borča d.o.o.; MP4—*Mentha piperita* from agricultural holding, Banatska Topola; OV1—*Origanum vulgare* from Institute for Medicinal Plant Research “Dr Josif Pancic”; SM3—*Satureja montana* from Bilje Borča d.o.o.; OM5—*Origanum majorana* from Geneza d.o.o.; TV6—*Thymus vulgaris* from agricultural holding, Kulpin; SH4—*Satureja hortensis* from agricultural holding, Banatska Topola; OB7—*Ocimum basilicum* from agricultural holding, Bačko Novo Selo; SO7—*Salvia officinalis* from agricultural holding, Bačko Novo Selo.

Comparisons of EOs’ extraction yields among Apiaceae, Asteraceae, Cupressaceae and Lamiaceae species are shown on Figure 1. The results on the Lamiaceae species presented in Figure 1d show that the highest yield of EOs was obtained by distillation of MP1 (2.15%), MP4 (1.32%) and SO7 (1.42%), while the lowest yields were obtained with OV3 (0.02%), OV1 (0.08%), SM3 (0.12%), MP2 (0.13%) and OM5 (0.26%). MP3 (0.62%), TV6 (0.99%), SH4 (0.71%) and OB7 (0.88%) had similar contents of EOs. The total yield of hydrodistillation of the Lamiaceae family has been extensively researched in recent years. Milojević et al. [21] reported EOs’ yields for different plants (and their parts). Data for sage flowers, leaves and stems (*Salvia officinilis*) had 1.8%, 2.0% and 0.4% of EOs, respectively; 3.1% for dried aboveground parts of summer savory (*Satureja hortensis)*; 0.7% for dried aboveground parts of winter savory (*Satureja montana*); 0.89% for fresh leaves of spearmint (*Mentha spicata*); 2.39% for dried aboveground parts of thyme (*Thymus vulgaris*) [21]. Abbas et al. [22] compared supercritical fluid extraction with the HD for basil EOs (*Ocimum basilicum* L.). The yield of EOs, obtained by HD, was 0.82%, which is comparable to the results in this work [22]. In the study by Bozin et al. [23], the EO yields of *Ocimum basilicum* L., *Origanum vulgare* L. and *Thymus vulgaris* L. were evaluated. The percentages of EO yields were as follows: *O. basilicum*, 0.37%; *O. vulgare*, 1.45%; and *T. vulgaris*, 1.80%. Ben Salha et al. [24] presented the work focused on *Origanum majorana* grown in Tunisia. The EO yield for dried marjoram aerial parts was 1.7%. 

Our samples showed that the obtained EO yields of the Asteraceae species were 0.29%, 0.05% and 0.01% in MC1, EP3 and EP2, respectively (Figure 1b). According to the literature, information about the genetic and environmental influences on EO yields, in similar plant parts used with HD, was available. In the study by Stanojević et al. [25], chamomile flowers collected from a plantation in the northwestern part of the Republic of Srpska, Bosnia and Herzegovina, were used for EOs’ isolation. Here, the yield of the obtained dark blue colored EOs was 0.5%. Aćimovic et al. [26] compared three different tetraploid chamomile cultivars from Coka (‘Zloty Lan’, ‘Manzana’ and ‘Lutea’). The EO contents ranged from 0.43 to 0.48% for all cultivars. On the other hand, in a review article by Sharifi-Rad et al. [27], it was stated that EO in Echinaceae plants contained 0.05–0.48% in fresh material and 0.1–1.25% in dried material. However, this amount may vary depending on the species. Furthermore, 1.85% for *E. purpurea* was reported in dry flowers and even less than 0.1% for *E. angustifolia* roots. These results are even higher than the values found in the research. Therefore, it could be concluded that EO in *Echinaceae purpurea* is found in floral heads, the amount of which depends on agroclimatic conditions.

The results of EOs’ yields for the Apiaceae family obtained from dried, ground seeds, located in the glands of the mericarp are shown in Figure 1a. From the graph, it can be seen that sample FV3 had the highest yield (5.80%), while the other samples (AG7, PC1, CC1 and PA1) had rather lower values (2.16%, 2.00%, 1.13% and 1.78%, respectively). As with other EOs, the literature lists numerous factors that affect their yields, e.g., origin of the seeds, maturity levels, environmental factors, etc. A recent review article by Sayed-Ahmad et al. [13] listed EO yields for different plants from Apiaceae species that were grown at different locations. Fennel, anise, dill and parsley from the Mediterranean region had the EO yields 3–6%, 2–6%, 1–4% and 2–8%, respectively, while caraway from Europe and Western Asia had 0.5–1.4% [13], which is consistent with the results from Figure 1a. 

The findings for EOs’ yields for the Cupressaceae family are shown in Figure 1c, for oils isolated from chopped dried fruits, as the berry fruits have elongated tubercles that serve as reservoirs for volatile oils. Here, herbal materials had similar moisture contents and particle diameters with different origins of juniper. To that end, samples were collected from three local facilities. EOs that came from the Institute for Medicinal Plant Research “Dr. Josif Pancic” had the highest yield of 1.90%, while samples from Adonis d.o.o., Sokobanja and Bilje Borča d.o.o., Borča had 1.33% and 1.52%, respectively. Our findings were in agreement with the literature data, where yields depended on the part of the plant used for HD. A review paper by Judžentienė [14] reported the main chemical compositions and EOs’ yields for different organs of Lithuanian *J. communis*. Here, the amounts of EOs varied drastically with maturity and plant anatomy, e.g., unripe berries had 2.5-times more EOs (0.3–4.2%) than the ripe ones (~0.9%). Leaves had 0.1–0.9% of EOs, while the contents of oil in sprouts and branches were very similar and equaled 0.2 and 0.16%, respectively [14].

### 2.2. Chemical Profile of EOs

The chemical constituents of EOs are mainly secondary metabolites whose content and profile are strongly dependent on the developmental stage, pedoclimatic conditions, drying, storing and type of used herbal materials [28]. The analysis of chemical components identified in the EOs of the Lamiaceae species confirmed that the oil consisted of several groups of components, namely, monoterpene hydrocarbons, oxygenated monoterpenes, sesquiterpene hydrocarbons and oxygenated sesquiterpenes. Identified compounds that constituted less than 0.1% EOs were referred to as trace components (tr). Table 2 shows the major components identified by the GC-MS in the Lamiaceae species’ EOs.

A total of 104 compounds were identified in the oils and their percentage ranged from 95.38% to 100%. Lamiaceae EOs are usually characterized by two or three major constituents [29]. The EOs of all samples contained mainly oxygenated monoterpenes with percentages ranging from 35.34% to 89.82%. The main components of this terpenoid group (>10%) were menthone (31.75% in MP1, 21.02% in MP2), menthol (37.63% in MP1, 33.54% in MP2), carvone (45.61% in MP3, 47.04% in MP4), *trans*-anethole (16.04% in OV1), geranyl acetate (12.64% in SM3), terpinen-4-ol (30.20% in OM5), thymol (55.65% in TV6), carvacrol (61.32% in SH4), linalool (24.62% in OB7) and camphor (26.48% in SO7). The differences in the concentration of menthol and menthone in the peppermint samples are possibly due to the maturity of the leaves and/or a specific harvesting stage. For instance, menthol is abundant in leaves harvested at the flowering stage, while younger leaves harvested at the bud stage contain relatively high levels of menthone [30]. A similar composition of oregano EOs was described in the article by Radusiene et al. [31], where variations in the percentages of compounds collected from different parts of herbal material were also reported (inflorescences and leaves). Carvacrol and thymol were detected only in small amounts (~1%), which is in line with the previously reported results. In a review article by Pateiro et al. [32], it was suggested that the application of EOs (oregano, thyme) in meat products prevented biochemical and microbial deterioration. The above-mentioned essential oils showed high antioxidant activity which could be attributed to the presence of thymol and carvacrol. A GC-MS analysis of the red thyme and summer savory EOs revealed that their antioxidant protection was nearly entirely due to the phenolic components, represented by thymol and carvacrol [33]. SM3, TV6, OM5, SH4 and SO7 also contained considerable amounts of monoterpene hydrocarbons (35.23%, 36.89%, 42.89%, 37.86% and 12.21%, respectively), with the most important being *p*-cymene (19.24% in SM3 and 25.04% in TV6), γ-terpinene (11.63% in OM5 and 29.14% in SH4) and camphene (5.33% in SO7). Sesquiterpene hydrocarbons accounted for about 8.56% and 42.06%. They were mainly represented by β-elemene (7.46% in OB7), *trans*-caryophyllene (12.54%, 5.69% and 14.60% in MP3, MP4 and OV1, respectively) and germacrene-D (14.56% in OV1). Oxygenated sesquiterpene was detected in smaller percentages (12.93% and 13.57%) in two samples (OV1 and OB7). They were expressed by caryophyllene oxide (8.82% in OV1) and τ-cadinol (10.93% in OB7). According to the literature data, the Karpiński review article listed a large group of plants belonging to the Lamiaceae family with their chemical compositions. The most abundant constituents in EOs include β-caryophyllene (41 plants), linalool (27 plants), limonene (26 plants), 1,8-cineole (22 plants), carvacrol (21 plants), α-pinene (21 plants), *p*-cymene (20 plants), γ-terpinene (20 plants) and thymol (20 plants), which are believed to have potent antifungal activity [34]. Similar constituents identified in the Lamiaceae species were included in the work of Ebadollahi et al. [8]. An overview of the anti-inflammatory effects of EOs by Zhao et al. [35], where a large group of plants from the Lamiaceae family are covered using in vitro and in vivo models, showed that different potential had been attributed to the varied EOs’ chemical structures. In this work, the same plants from the Lamiaceae family have been studied, such as thyme, peppermint, oregano and sage, which possess oxygenated monoterpenes (menthol, menthone, carvacrol, thymol, camphor, geraniol) as the dominant constituents.

The main constituents of EOs from the Asteraceae species were terpenes (Table 3). In this case, the chemical structure of a chamomile sample was analyzed (MC1).

A total of 19 components were identified from the EOs by the GC-MS analysis, accounting for 97.01% of all detected molecular species. A-Bisabolol oxide A (30.64%) was the main component, followed by α-bisabolol oxide B (19.93%), α-bisabolol (6.17%) and α-bisabolone oxide A (4.57%), which belonged to the main group of oxygenated sesquiterpenes (63.26%). Other compounds (sesquiterpenes hydrocarbons) accounted for 10.49% of the total oil and consisted mainly of chamazulene (5.54%) and *trans*-β-farnesene (4.84%). Polyacetylene compounds, called spiroethers, were detected in two unequally represented isomeric forms: *trans*-spiroethers (0.44%) and *cis*-spiroethers (22.10%). 

Sesquiterpene compounds are considered to be the most important bioactive compounds in chamomile, providing a rich spectrum of aromas that are usually found to be very pleasant [10]. According to Sharifi-Rad et al. [36], there are wide variations in the qualitative and quantitative compositions of volatile fractions obtained from chamomiles originated from different countries and regions. However, the main components are sesquiterpenes such as (-)-α-bisabolol (5–70%), bisabolol oxide A (5–60%), bisabolol oxide B (5–60%), bisabolone oxide (0–8%), (E)-β-farnesene (7–45%) and chamazulene (1–35%) [36]. Tirillini et al. [37] identified more than 70 components (accounting for about 99% of chamomile EOs), isolated from tubular ligulate flowers and receptacles. The predominant groups of EOs were the oxygenated sesquiterpenes (42%, 28.8% and 44.9%, respectively), the sesquiterpene hydrocarbons (40.1%, 40.7% and 35.7%, respectively) and the ether fractions (2.8%, 13.7% and 6.5%, respectively) [37]. In a research paper by Wang et al. [38], three types of chamomiles (German chamomile, Roman chamomile and Juhua) were analyzed. The obtained results showed that the first one had a similar GC-MS profile as the chamomile sample from this research (predominant constituents were α-bisabolol oxide B, α-bisabolol, α-bisabolol oxide A and farnesene), while the other two had monoterpene hydrocarbons and oxygenated monoterpenes as dominant components.

Various volatiles were present in EOs from the Apiaceae family (extracted from seeds/fruits). The result of the GC-MS analysis is shown in Table 4.

A total of 41 compounds were identified in the five samples. The percentages of the identified components varied from 98.71% in PA1 to 100% in the others. The predominant compound found in PA1 and FV7 belonged to the group of oxygenated monoterpenes (98.58% and 93.55%, respectively). Furthermore, *trans*-Anethole was detected as 96.40% in PA1 and 73.85% in FV7, and it was also responsible for the characteristic anise aroma and taste. In samples CC1 and AG7, most of the citrus aroma was derived from limonene (27.63% and 45.24%, respectively), which belonged to the group of monoterpene hydrocarbon (27.63% and 49.68%, respectively). The two previously mentioned samples contained another important flavoring and fragrance originated from carvone (70.25% and 45.90%, respectively), which belonged to the group of oxygenated monoterpenes (72.29% and 50.32%, respectively). The PC1 sample, on the other hand, had a completely different structure. Phenylpropanoids, such as myristicin, elemicin, 6-methoxyelemicin and apiole, occurred in a range from 5.53% to 35.81%. Furthermore, being extracted in these tested samples from fruit or seeds, such compounds can be also extracted from the roots.

Chizzola [39] reviewed major constituents in EOs from wild plants belonging to the European Apiaceae species grown in the Mediterranean region, including Asia Minor [39]. It was concluded in the report that, in addition to the origins of the plants, the composition of the oils in different plant organs of the same species can be quite different. Accordingly, the fruit oil of Laser trilobum was dominated by limonene and perillaldehyde, while the oil from the leaves of the same plant contained bornyl acetate as its main component. Root oils may differ from the oils of the aerial parts, for example, in Daucus carota ssp maxima, where the root contained the phenylpropanoids, dill apiole and myristicin, and the inflorescences contained mainly sabinene and terpinen-4-ol [39]. A review paper by Spinozzi et al. [40] listed a large group of plants from this family. EO is hydrodistillated from different plant parts, such as schizocarps, herbs and aerial parts, but the GC-MS profiles are relevant to the data obtained in this research. Besides their major EO constituents, this work presented the utilization of those products as botanical insecticides against mosquitos. Even if these eco-friendly insecticides show many advantages, the main issue to overcome is the low persistence of their effect, due to their volatile compounds. 

A chemical analysis which resulted from the detection of 40 compounds in the Cupressaceae family’s EOs is shown in Table 5. This table lists the identified compounds, which accounted for 97.92% of the composition of the total extracts.

Monoterpene hydrocarbons were the predominant group of compounds in all samples and were found to be between 64.96% in JC2, 69.24% in JC1 and 70.27% in JC3 of the content, followed by sesquiterpene hydrocarbons (26.04% in JC2, 23.70% in JC1 to 21.74% in JC3). In all samples, two components, *ɑ*-pinene and sabinene, accounted for an average of 35.79% and 14.86%, respectively. Fourteen compounds, including limonene, β-elemene, *trans*-caryophyllene, γ-elemene, germacrene D and δ-cadinene, were present in moderate to high quantities (1.5–8.0%). From these analyses, it could be concluded that all samples had similar chemical compositions, and that the distributions of compounds were similar. 

In the study by Rajčević et al. [41], the aim was to determine the diversity of terpene classes and common terpenes in populations of typical *Juniperus communis* from the northwestern Balkans (Slovenia and Croatia) grown under different climate conditions, on different substrates and at different altitudes. These samples showed the dominance of the chemotypes sabinene and *ɑ*-pinene in the composition of the EOs’ profiles. However, the reported EOs’ contents showed much greater variability between the alpine and continental populations. Continental populations, with over 10 different compounds (including limonene, β-phellandrene, (*E*)-caryophyllene, β-copaene, germacrene D and germacrene B) were present in high amounts (>6.0%) [41].

**Figure 2:**
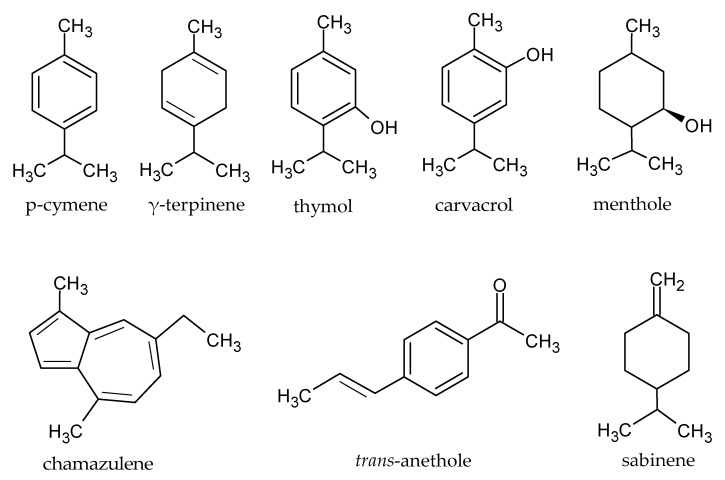
The chemical structures of major constituents identified by GC-MS analysis from studied families.

The chemical structures of major constituents identified by the GC-MS analysis of the different above-mentioned families are shown in Figure 2.

### 2.3. In Vitro Antioxidant Activity

The in vitro antioxidant activity of EOs was evaluated by assessing their ability to bind hydrogen or scavenge radicals using DPPH and ABTS^+^. The study of antioxidants is a hot topic because they play an important role in preventing cellular damages caused by free radicals, leading to various degenerative diseases in humans such as cancer, heart diseases and others [42]. The changes in antioxidant capacity affected by different EOs from four families are shown in Table 6.

The MC1 EO was the most effective DPPH radical scavenger (44.20 µM Trolox/g) at a tested concentration of 10 mg/mL, followed by samples from the Lamiaceae family such as TV6 (29.78 µM Trolox/g at 10 mg/mL), SH4 (23.32 µM Trolox/g at 10 mg/mL) and OB7 (44.97 µM Trolox/g at 15 mg/mL). The high antioxidant capacity of chamomile inflorescence was also reported by a review from Petronilho who attributed it to the presence of chamazulene and α-bisabolol [43].

Thymol, carvacrol, linalool and estragole were the main contributors to the antioxidant activity of the volatile extracts of thyme, summer savory and basil, as noted in a previous study [42]. The effects of the other samples were significantly different depending on the tested concentration. Accordingly, the samples of the Apiaceae species showed lower DPPH activity than the EOs of AG7 and FV7 with a value of 2.28 µM Trolox/g and 1.37 µM Trolox/g at 10 mg/mL, respectively. The antioxidant activity of the Apiaceae family was reported for extracts and EOs, with higher activity observed in extracts due to the presence of phenolic compounds, such as flavonoids and proanthocyanidins, carotenoids, etc. [13]. The study by Hajlaoui et al. [44] displayed the moderate DPPH scavenging activity of *C. carvi* and *C. sativum* EOs and their mixtures, due to the presence of γ-terpinene, carvone, linalool and *p*-cymene as dominant terpenes. The EOs rich in this group of monoterpenes increased the oxidative stability and shelf life of edible lipids, through their interactions with other monoterpenes, rather than single antioxidants. Samples obtained by the distillation of MP1-MP4 showed weak scavenging ability for the DPPH radicals (average 5.49 µM Trolox/g at 15 and 20 mg/mL). This was in agreement with the literature data which reported that peppermint terpenoids have modest activity in the model system [45]. Samples from the Cupressaceae species showed a good result for DPPH activity at 15 mg/mL (average 3.50 µM Trolox/g). However, the low DPPH activity of juniper berry EO may be due to the high amount of α-pinene and β-pinene, both of which were inactive in the DPPH test, as was concluded in the research paper by Emami et al. [46].

The antioxidant activity of the ABTS^+^ assay improved dramatically for EOs derived from a large group of Lamiaceae and Cupressaceae families. This was consistent with our previous results where we also reported that peppermint EO had higher activity towards ABTS^+^ as compared to DPPH radicals [47]. The activity of most samples from the Lamiaceae family ranged from 296.69 µM Trolox/g (MP3) to 757.98 µM Trolox/g (OB7) at a concentration of 1 mg/mL. At the same concentration, the samples from the Asteraceae family reached a high activity of 406.56 µM Trolox/g on average, similar to the samples from the Cupressaceae family, where the average activity was 308.06 µM Trolox/g. The Apiaceae family showed the lowest ABTS^+^ radical scavenging activity: even at a ten-fold higher concentration it was still 34.44 µM Trolox/g. Similarly, Hoferl et al. [48] observed that juniper berry EO showed a significant inhibitory effect on ABTS^+^ radicals (IC_50_ 10.96 µg/mL), but butylated hydroxytoluene was considerably stronger (IC_50_ 0.0175 µg/mL).

This work provides a preliminary study with in-depth information of the antioxidant potential of the most common EOs obtained from aromatic plants grown in Serbia. This will be used as a screening tool for the selection of further EO candidates for further processing by emerging extraction techniques such as microwave-assisted hydrodistillation and supercritical fluid extraction. Our previous studies suggested that selected EOs obtained from sage [49], winter savory [50] and wild thyme [51] could be efficiently used as natural additives with antioxidant and antimicrobial properties in various meat products [6,52]. Furthermore, coriander EO was efficiently used as a partial nitrite replacement in meat products, thus suggesting that natural extracts could diminish the concentration of potentially toxic additives in food products [53]. It could be observed that the majority of these EOs were obtained from the plant species from the Lamiaceae family. However, plants from the Apiaceae family had the highest content of EO among the investigated plant species. These EOs were already recognized with a broad spectra of applications as potent antioxidants in food, nutraceuticals and cosmetics [54]. Based on this, our further research on the application of EOs as natural additives will be focused on the Apiaceae family, particularly on dill and caraway essential oils.

In conclusion, the samples which turned out to be promising as potential antioxidants were the ones from the Asteraceae family (the most effective DPPH radical scavengers), while the samples from the Lamiaceae and Cupressaceae families showed high antioxidant activity against ABTS^+^ radicals.

### 2.4. Principal Component Analysis (PCA)

**Figure 3:**
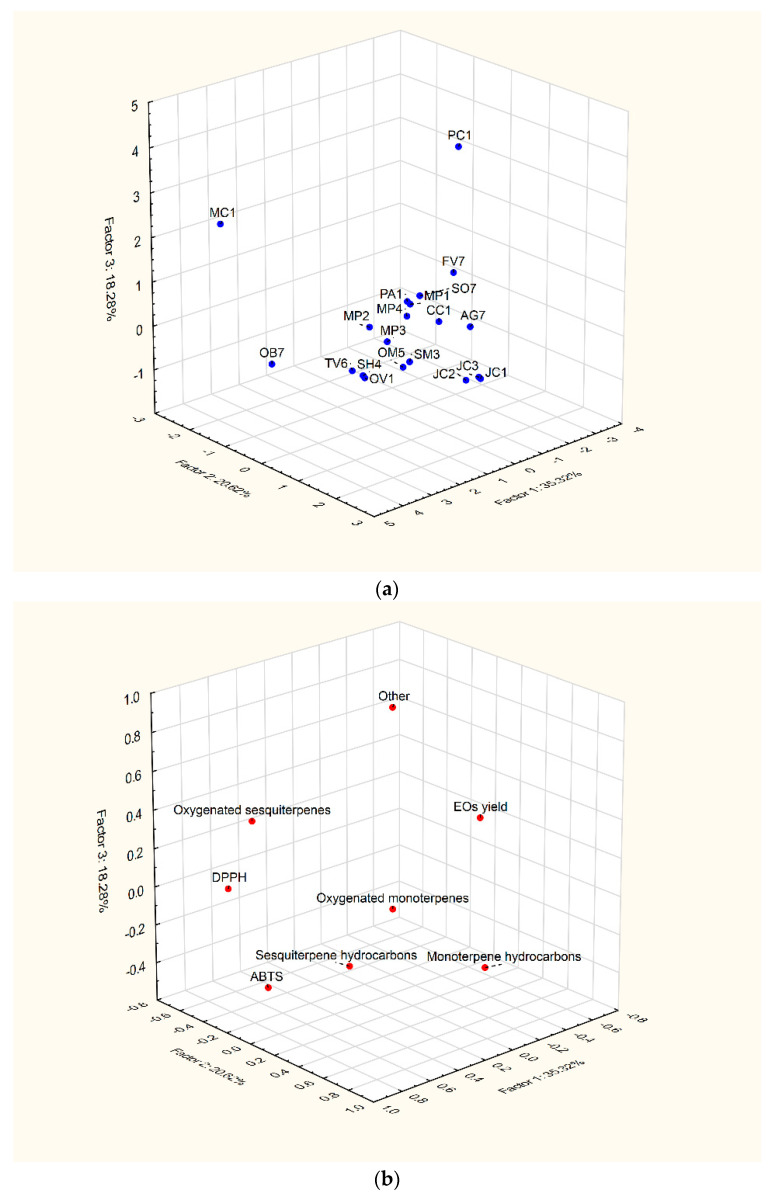
PCA loading plots in the plane of factors 2 and 3 vs. factor 1: (**a**) the labels correspond to samples, and (**b**) the labels correspond to variables.

A PCA was used to describe the differences between samples and to provide more information on the response variables that mainly influenced sample similarities and differences, in order to reduce the complexity of the data and address the most important features [55]. In this study, the application of the PCA on the dataset was used to analyze the antioxidant potential (DPPH and ABTS^+^) of the evaluated EOs from different plant samples, as well as their yields and chemical profiles. As indicated in Table 7 and shown in Figure 3, according to the results of the PCA and Kaiser’s rule, the first three PCs explained 74.2% of the total variance of the experimental data. The factor coordinates of all samples and variables are presented in Table S1 (Supplementary Materials).

The first PC (F1) was mainly associated with EOs’ yield, antioxidant activity and sesquiterpenes. Samples MC1 and OB7, followed by the OV1 sample, were allocated at the positive side of F1, near oxygenated sesquiterpenes, DPPH and ABTS, thus indicating an increasing number of bioactive components such as β-elemene, τ-cadinol, α-bisabolol oxide A and α-bisabolol oxide B (sesquiterpenes) responsible for their high antioxidant potential. The negative side of F1 was related to sample FV7, which was characterized by the highest EO yield. On the other hand, according to the factor coordinates of samples and variables, based on correlations, the samples MC1, OB7 and OV1 showed the lowest EO yield. It was also observed that oxygenated monoterpenes contributed significantly to both the first and the second PCA. Therefore, samples FV7 and PA1 were strongly associated with oxygenated monoterpenes.

The second PC (F2) was mainly associated with monoterpene hydrocarbons and showed a clear difference between a group of samples from the Cuppresaceae family (JC1, JC2 and JC3) and all other samples, as they received the highest composition of monoterpene hydrocarbons, such as α-pinene, sabinene, myrcene and limonene, and a very low proportion of oxygenated monoterpenes. This chemical structure could represent those samples as promising agents against ABTS^+^ radicals. On the other hand, a great group of samples from Apiaceae (FV7, PA1) and Lamiaceae (MP1, MP2, MP4) had dominant chemical compositions of oxygenated monoterpenes.

In addition, the third PC (F3) was associated with other chemical constituents important for the characterization of the PC1 sample, followed by the MC1 sample. The major other components were myristicin, elemicin, 6-methoxyelemicin and apiol. Since these components were mainly found in the PC1 sample, they could be a reason for the low antioxidant potential of this sample. The other plant samples were grouped, according to F3 coordinate values, indicating the small amount of other chemical constituents (<8%). 

## 3. Materials and Methods

### 3.1. Plant Material

The plant material was collected from different places in Serbia. Some of them were grown in agricultural holdings in Bačko Novo Selo (basil, sage, fennel and dill), Kulpin (thyme) and Banatska Topola (peppermint and summer savory). Harvesting was completed by hand at the stage of full maturity, in the summer of 2019. After harvesting, the plant material was stored in paper bags at room temperature until the need for further analysis. The other plants were purchased from local institutions: the Institute for Medicinal Plant Research “Dr Josif Pancic” (peppermint, oregano, chamomile, parsley, caraway, anise and juniper); medicinal and aromatic herbs and herb-based products, Adonis d.o.o., Sokobanja (peppermint, echinacea and juniper); medicinal and aromatic herbs and herbal-based products, Bilje Borča Llc, Borča (peppermint, oregano, winter savory, echinacea and juniper); medicinal and aromatic herbs and herbal products, Geneza d.o.o., Kanjiza (marjoram). The dried plant material was grounded in a household blender (except for those that were purchased as grounded), while the particle size of the material was determined by sieve sets (CISA, Cedaceria Industrial, Barcelona, Spain). The moisture content of the plant material was analyzed using a standard procedure, i.e., by drying the plant sample at 110 °C until reaching a constant weight. All codes for the samples and characterizations of the plants are shown in Table 1.

### 3.2. Chemicals

The standard compounds used for the GC analysis were thymol, *trans*-anethol, (+)-borneol, (−)-borneol, α-terpineol, L-carvone, (R)-(+)-limonene, eucalyptol, farnesol, neryl acetate, (±)-citronellal, citral, γ-terpinene, nerol, α-pinene, *p*-cymene, (−)-*trans*-caryophyllene, geraniol, geranyl acetate, carvacrol, eugenol, sabinene hydrate, bornyl acetate, linalyl acetate, myrcene and (±)-camphor and were purchased from Sigma-Aldrich (St. Louis, MO, USA). In addition, 1,1-Diphenyl-2-picryl-hydrazyl-hydrate (DPPH∙) was obtained from Sigma-Aldrich GmbH (Germany). All chemicals and solvents were analytical reagent grade.

### 3.3. Isolation of EO—Conventional Hydrodistillation (HD)

Clevenger’s hydrodistillation was used for the isolation of EOs, i.e., slightly modified official *Ph*. *Eur*. VII Procedure [56]. Forty grams of the milled, dried plant material was placed in a round glass flask (1 L), and 400 mL of distilled water was added. Distillation was carried out for 2 h and it was performed in triplicates. The EO yield (Y) was expressed as % (*v*/*w*). Samples were collected and stored at 4 °C in dark bottles, to avoid deterioration before further analysis.

### 3.4. Gas Chromatography-Mass Spectrometry (GC–MS) Analysis

The chemical composition of EOs obtained by HD was analyzed by GC system (7890A, Agilent Technologies, Santa Clara, CA, USA) coupled with MS (5975C, Agilent Technologies, Santa Clara, CA, USA) and HP-5MSUI (30 m × 0.25 mm, 0.25 µm) capillary column. The EOs were dissolved in methylene chloride, and 2 µL of the properly diluted samples was added to the GC via TriPlus AS autosampler. The mobile phase was helium (>99.9997%) at a flow rate of 2 mL/min. The temperature program was as follows: the initial temperature was 45 °C (8 min), then increased to 230 °C at a rate of 8.0 °C/min and remained at this temperature for 10 min. The injector, mass transfer line and ion source temperature were 250, 200 and 220 °C, respectively. Compounds were identified using the NIST database of MS spectra and the database in the literature [57], with the final results reported as relative content (%). For additional confirmation, the linear retention indices were calculated and compared with the existing literature [57]. A quantitative analysis was performed by generating the calibration curves for the analyzed compound in the concentration range of 1–500 µg/mL. 

### 3.5. In Vitro Antioxidant Activity

The capacity of EO samples, in terms of hydrogen donating or radical scavenging ability, was measured using the stable radical DPPH according to the published method [58] with slight modifications for lipid samples [59]. For this purpose, a methanolic solution of DPPH reagent (65 µM) was freshly prepared and adjusted to an absorbance of 0.70 (±0.02) with methanol, and 0.1 mL of the samples diluted in ethyl acetate was added to 2.9 mL of DPPH reagent. Blank samples were prepared by mixing 0.1 mL of ethyl acetate and 2.9 mL of DPPH reagent. Samples were stored at room temperature and in the dark for 60 min. Free radical scavenging measurements were performed in triplicates at a wavelength of 517 nm by UV/Vis spectrophotometer (6300 Spectrophotometer, Jenway, Staffordshire, UK). The obtained results were expressed as µM Trolox equivalents per g EO (µM Trolox/g). As is represented in Table 5, the concentrations of essential oils were 10, 15 and 20 mg/mL.

The ability of the samples to scavenge ABTS^+^ radicals was measured using a modified method from the literature [60]. Briefly, the ABTS stock solution was freshly prepared from a mixture (1:1, *v*/*v*) of 2.45 mM potassium persulphate aqueous solution and 7 mM ABTS aqueous solution, and then allowed to stand in the dark at room temperature for 16 h. The stock solution was diluted with absolute ethanol to achieve an absorbance of 0.70 (±0.02). A volume of 0.1 mL of the properly diluted EO samples in ethyl acetate and 2.9 mL of the ABTS reagent were mixed and incubated for 5 h in the dark at room temperature. The blank samples were obtained by mixing 0.1 mL ethyl acetate and 2.9 mL ABTS reagent. Absorbance was measured at 734 nm in triplicates using a UV/Vis spectrophotometer (6300 Spectrophotometer, Jenway, Staffordshire, UK). The results were expressed as µM Trolox equivalents per g EO (µM Trolox/g), as mean ± standard deviation. As is represented in Table 5, the concentrations of essential oils were 1 and 10 mg/mL.

### 3.6. Statistical Analysis and PCA

All experiments were performed in triplicates. A statistical analysis was performed using the Statistica version 8 software package. A PCA calculation was performed to obtain an insight into the relationships between data obtained by the EO yield, chemical properties (compounds with abundance higher than 1%) and antioxidant activity (DPPH and ABTS assays).

## 4. Conclusions

Considering the high level of public awareness of the beneficial influences of natural bioactives for the food and pharmaceutical industries, alternative medicine and natural therapy, and in conjunction with the higher content of terpene compounds in various members of the Lamiaceae, Asteraceae, Apiaceae and Cupressaceae species, different medicinal and aromatic plants from Serbia were hydrodistilled in this work as a possible source of high-potency bioactives. 

Generally, the highest EOs’ yields were detected for dried, milled seeds of the Apiaceae family, which were dominated by the group of oxygenated monoterpenes (carvone and *trans*-anethole). Due to the high content of sesquiterpenes, dominated by α-bisabolol oxide A and α-bisabolol oxide B, the samples from the Asteraceae family were the most effective DPPH radical scavengers; however, the samples from the Lamiaceae and Cupressaceae families showed high antioxidant activity against ABTS^+^ radicals, dominated by monoterpenes, where the main components of this terpenoid group were menthone, menthol, carvone, thymol, carvacrol and α-pinene.

This work provided in-depth information on the antioxidant potentials for the most common EOs obtained from aromatic plants commonly present in Serbia. The reported data can be used as a screening tool for further selection of EOs’ candidates to be industrially exploited (microwave-assisted, supercritical fluid extractions, etc.) and processed with other types of advanced extractions. One of the industrial applications can be for the food industry, as we suggested previously for sage [49], winter savory [50], wild thyme [51] and coriander [53]. EOs from these plants can be efficiently used as natural additives with antioxidant and antimicrobial properties for various meat products [52]. From our and other available results, it can be noticed that the majority of these EOs were obtained from the Lamiaceae family. However, based on the EOs’ yields, chemical profiles and in vitro antioxidant activities, further research will be focused on the Apiaceae family, particularly dill and caraway’s essential oils.

## Figures and Tables

**Table 1:** Plant material data.

Family	Plant Name English	Plant Name Latin	Analyzed Part of the Plant	Sample Name	Plant Properties	
					MC [%, *w*:*w*]	d [mm]
Apiaceae	Dill	*Anethum graveolens*	Chopped fruit/seed	AG7	8.49	0.3358
	Caraway	*Carum carvi*	Chopped fruit/seed	CC1	9.86	0.9163
	Fennel	*Foeniculum vulgare*	Chopped fruit/seed	FV7	9.96	0.6695
	Parsley	*Petroselinum crispum*	Chopped fruit/seed	PC1	9.63	0.9525
	Anise	*Pimpinella anisum*	Chopped fruit/seed	PA1	8.18	0.7563
Asteraceae	Echinacea	*Ehinacea purpurea*	Chopped herb	EP2	10.25	0.9784
			Chopped herb	EP3	10.74	0.898
	Chamomile	*Matricaria chamomilla*	Chopped flower	MC1	7.84	3.5930
Cupressaceae	Juniper	*Juniperus comunis*	Chopped fruit	JC1	10.98	0.9686
			Chopped fruit	JC2	10.29	0.8714
			Chopped fruit	JC3	10.65	0.9716
Lamiaceae	Peppermint	*Mentha x Piperita*	Pharmacologically crushed leaf	MP1	13.02	1.5920
			Chopped herb	MP2	9.58	1.0260
			Chopped leaf	MP3	9.48	1.9045
			Chopped leaf	MP4	10.52	/
	Basil	*Ocimum basilicum*	Chopped herb	OB7	8.38	0.5493
	Marjoram	*Origanum majorana*	Chopped leaf	OM5	8.02	0.8218
	Oregano	*Origanum vulgare*	Chopped herb	OV1	0.00	0.9213
			Chopped herb	OV3	8.39	0.7688
	Sage	*Salvia officinalis*	Chopped herb	SO7	9.95	1.5928
	Winter savory	*Satureja montana*	Chopped herb	SM3	10.91	0.9342
	Summer savory	*Satureja hortensis*	Chopped herb	SH4	12.22	/
	Thyme	*Thymus vulgaris*	Chopped herb	TV6	11.19	0.4426

AG7—*Anethum graveolens* from agricultural holding, Bačko Novo Selo; CC1—*Carum carvi* from Institute from Medicinal Plant Research “Dr Josif Pancic”; FV7—*Foeniculum vulgare* from agricultural holding, Bačko Novo Selo; PC1—*Petroselinum crispum* from Medicinal Plant Research “Dr Josif Pancic”; PA1—*Pimpinella anisum* from Institute for Medicinal Plant Research “Dr Josif Pancic”; EP2—*Ehinacea purpurea* from Adonis d.o.o.; EP3—*Ehinacea purpurea* from Bilje Borča d.o.o.; MC1—*Matricaria chamomilla* from Institute for Medicinal Plant Research “Dr Josif Pancic”; JC2—*Juniperus comunis* from Adonis d.o.o.; JC3—*Juniperus comunis* from Bilje Borča d.o.o.; JC1—*Juniperus comunis* from Institute for Medicinal Plant Research “Dr Josif Pancic”; MP1—*Mentha piperita* from Institute for Medicinal Plant Research “Dr Josif Pancic”; MP2—*Mentha piperita* from Adonis d.o.o.; MP3—*Mentha piperita* from Bilje Borča d.o.o.; MP4—*Mentha piperita* from agricultural holding, Banatska Topola; OB7—*Ocimum basilicum* from agricultural holding, Bačko Novo Selo; OM5—*Origanum majorana* from Geneza d.o.o.; OV1—*Origanum vulgare* from Institute for Medicinal Plant Research “Dr Josif Pancic”; OV3—*Origanum vulgare* from Bilje Borča d.o.o.; SO7—*Salvia officinalis* from agricultural holding, Bačko Novo Selo; SM3—*Satureja montana* from Bilje Borča d.o.o.; SH4—*Satureja hortensis* from agricultural holding, Banatska Topola; TV6—*Thymus vulgaris* from agricultural holding, Kulpin; MC—moisture content; d—particle size.

**Table 2:** GC-MS profile of EOs obtained from Lamiaceae species.

No	Component	RT ^[a]^	Relative Percentage (%)										
			MP1	MP2	MP3	MP4	OV1	SM3	OM5	TV6	SH4	OB7	SO7
1	α-Thujene	3.8	ND	ND	ND	ND	tr	ND	0.69	0.64	0.64	ND	ND
2	α-Pinene	3.9	0.34	0.19	0.43	0.49	0.13	2.15	0.58	0.81	0.29	ND	2.93
3	Camphene	4.2	ND	ND	ND	ND	0.13	0.73	ND	0.23	tr	ND	5.33
4	Sabinene	4.8	0.13	tr	tr	0.17	1.66	0.28	3.44	tr	ND	tr	tr
5	β-Pinene	4.9	0.76	0.29	0.87	0.88	ND	ND	ND	tr	0.10	ND	1.77
6	1-Octen-3-ol	5.0	ND	ND	ND	ND	0.49	0.27	ND	ND	ND	ND	ND
7	Myrcene	5.2	tr	0.12	0.34	1.61	0.28	0.36	0.73	0.84	0.91	0.14	0.42
8	α-Phellandrene	5.5	ND	ND	ND	ND	ND	0.11	0.60	ND	ND	ND	ND
9	α-Terpinene	5.8	ND	0.17	ND	ND	0.23	0.40	7.37	1.00	2.49	ND	0.15
10	*p*-Cymene	6.1	ND	ND	ND	ND	1.91	19.24	7.47	25.04	4.29	ND	0.09
11	Limonene	6.2	0.26	0.37	0.91	3.86	ND	7.83	3.23	ND	ND	tr	0.82
12	β-Phellandrene	6.2	ND	ND	ND	ND	ND	ND	4.64	ND	ND	ND	ND
13	Eucalyptol (1,8-Cineole)	6.2	6.56	3.62	8.27	11.19	1.64	ND	ND	ND	ND	1.83	7.27
14	*cis*-β-Ocimene	6.5	ND	ND	ND	ND	0.70	0.94	ND	ND	ND	ND	ND
15	*trans*-β-Ocimene	6.8	tr	ND	ND	0.17	0.23	0.93	ND	ND	tr	0.38	ND
16	γ-Terpinene	7.1	0.13	0.37	tr	ND	0.49	1.74	11.63	8.20	29.14	ND	0.28
17	*cis*-Sabinene hydrate	7.4	0.75	0.35	0.14	0.12	ND	0.72	1.49	0.69	tr	ND	0.10
18	*cis*-Linalool oxide (furanoid)	7.6	ND	ND	ND	ND	ND	0.36	ND	ND	ND	tr	ND
19	Terpinolene	8.0	tr	0.14	ND	ND	ND	0.19	2.52	0.13	tr	ND	0.42
20	Fenchone	8.0	ND	ND	ND	ND	0.67	ND	ND	ND	ND	0.18	ND
21	*trans*-Linalool oxide (furanoid)	8.1	ND	ND	ND	ND	ND	0.18	ND	ND	ND	ND	ND
22	*trans*-Sabinene hydrate	8.4	ND	ND	ND	ND	ND	ND	3.44	ND	ND	ND	0.07
23	Linalool	8.4	0.19	0.65	0.10	0.15	3.30	11.91	3.27	1.97	tr	24.62	0.12
24	Isoamyl isovalerate	8.5	tr	ND	0.27	0.41	ND	ND	ND	ND	ND	ND	ND
25	α-Thujone	8.6	ND	ND	ND	ND	0.88	ND	ND	ND	ND	ND	25.80
26	β-Thujone	9.0	ND	ND	ND	ND	0.08	ND	ND	ND	ND	ND	6.20
27	*cis*-Menth-2-en-1-ol	9.2	ND	0.16	ND	ND	ND	0.21	2.20	ND	ND	ND	ND
28	allo-Ocimene	9.5	ND	ND	ND	ND	0.11	0.11	ND	ND	ND	ND	ND
29	*trans*-Menth-2-en-1-ol	9.9	ND	ND	ND	ND	ND	ND	1.46	ND	ND	ND	ND
30	Camphor	9.9	ND	0.14	ND	ND	0.35	0.33	ND	0.35	ND	1.13	26.48
31	Menthone	10.3	31.75	21.02	2.88	7.05	0.84	0.29	ND	ND	ND	ND	ND
32	Isomenthone	10.7	6.55	4.05	0.64	1.82	0.12	ND	ND	ND	ND	ND	ND
33	Neomenthol	10.8	3.58	2.71	tr	0.85	ND	ND	ND	ND	ND	ND	ND
34	Borneol	10.8	ND	ND	ND	ND	0.77	5.29	tr	1.11	tr	tr	2.26
35	δ-Terpineol	11.0	ND	ND	ND	ND	ND	ND	ND	ND	ND	tr	ND
36	Menthol	11.1	37.63	33.54	6.30	7.94	0.79	ND	ND	ND	ND	ND	ND
37	Terpinen-4-ol	11.3	ND	ND	ND	ND	0.92	2.44	30.20	0.61	0.18	tr	0.19
38	Isomenthol	11.5	0.40	0.75	0.10	0.16	ND	ND	ND	ND	ND	ND	ND
39	Neoisomenthol	11.7	ND	tr	ND	ND	ND	ND	ND	ND	ND	ND	ND
40	α-Terpineol	11.8	tr	0.13	0.16	ND	4.55	0.50	3.75	tr	ND	0.25	ND
41	*cis*-Dihydrocarvone	12.0	ND	1.11	3.01	4.38	ND	0.31	ND	ND	ND	ND	ND
42	*cis*-Piperitol	12.0	ND	ND	ND	ND	ND	ND	0.97	ND	ND	ND	ND
43	Estragole	12.1	ND	ND	ND	ND	0.68	ND	0.77	ND	ND	16.99	ND
44	*trans*-Dihydrocarvone	12.3	ND	ND	0.25	0.24	ND	0.47	ND	ND	ND	ND	ND
45	*trans*-Piperitol	12.5	ND	ND	ND	ND	ND	ND	0.50	ND	ND	ND	ND
46	*trans*-Carveol	13.0	ND	0.25	tr	tr	ND	0.35	ND	ND	ND	ND	ND
47	Nerol	13.3	ND	ND	ND	ND	ND	0.36	ND	ND	ND	ND	ND
48	Thymol methyl ether	13.5	ND	ND	ND	ND	0.14	0.23	ND	0.10	ND	ND	ND
49	*cis*-3-Hexenyl isovalerate	13.5	ND	ND	0.16	0.19	ND	ND	ND	ND	ND	ND	ND
50	Pulegone	13.7	1.74	1.26	0.17	0.41	0.11	ND	ND	ND	ND	ND	ND
51	Neral (Citral)	13.8	ND	ND	ND	ND	ND	0.21	ND	ND	ND	ND	ND
52	Carvone	13.9	ND	10.96	45.61	47.04	0.62	1.11	1.06	ND	ND	0.12	ND
53	Piperitone	14.3	0.67	1.97	1.04	0.22	ND	ND	ND	ND	ND	ND	ND
54	Linalool acetate	14.3	ND	ND	ND	ND	0.64	ND	0.70	ND	ND	ND	ND
55	Geraniol	14.4	ND	ND	ND	ND	ND	10.15	ND	ND	ND	0.51	ND
56	Geranial	15.0	ND	ND	ND	ND	ND	0.41	ND	ND	ND	ND	ND
57	*trans*-Carane	15.0	0.19	0.21	tr	tr	ND	ND	ND	ND	ND	ND	ND
58	Bornyl acetate	15.4	ND	ND	ND	ND	0.72	0.39	0.09	ND	ND	0.48	1.11
59	Dihydroedulane I	15.5	tr	1.07	0.45	0.13	ND	ND	ND	ND	ND	ND	ND
60	Dihydroedulane I	15.7	ND	0.76	0.58	0.20	ND	ND	ND	ND	ND	ND	ND
61	*trans*-Anethole	15.6	ND	ND	ND	ND	16.04	ND	ND	ND	ND	3.09	ND
62	*cis*-Carane	15.8	4.25	3.51	1.18	0.82	ND	ND	ND	ND	ND	ND	ND
63	Thymol derivative	16.2	ND	ND	ND	ND	ND	ND	ND	0.24	ND	ND	ND
64	Menthyl acetate	16.4	0.20	0.17	ND	tr	ND	ND	ND	ND	ND	ND	ND
65	Thymol	16.6	ND	ND	ND	ND	0.44	0.43	ND	55.65	tr	ND	ND
66	Carvacrol	16.9	ND	ND	ND	ND	1.19	ND	0.43	ND	61.32	ND	ND
67	1,5,5-Trimethyl-6-methylene-cyclohexene	17.4	ND	tr	0.30	0.12	ND	0.22	ND	ND	ND	0.35	ND
68	α-Cubebene	18.0	ND	ND	ND	ND	ND	ND	ND	ND	ND	0.13	ND
69	α-Terpinyl acetate	18.1	ND	ND	ND	ND	ND	0.86	ND	ND	ND	ND	ND
70	Eugenol	18.7	ND	ND	ND	ND	ND	ND	ND	ND	ND	ND	ND
71	α-Copaene	19.0	ND	ND	ND	ND	0.49	0.22	0.16	ND	ND	0.46	ND
72	β-Bourbonene	19.3	0.16	0.43	1.85	1.16	1.48	1.53	ND	ND	ND	0.48	ND
73	Geranyl acetate	19.5	ND	ND	ND	ND	ND	12.64	ND	ND	ND	ND	ND
74	β-Cubebene	19.6	ND	ND	ND	ND	ND	ND	ND	ND	ND	0.13	ND
75	β-Elemene	19.7	0.04	tr	0.27	tr	0.46	ND	ND	ND	ND	7.46	ND
76	*cis*-Caryophyllene	20.2	ND	ND	ND	tr	ND	ND	ND	ND	ND	ND	ND
77	*trans*-Caryophyllene	20.6	1.63	tr	12.54	5.69	14.60	2.50	2.74	0.97	ND	0.98	1.26
78	β-Caryophyllene	20.7	ND	ND	ND	ND	ND	ND	ND	ND	0.52	ND	ND
79	β-Gurjunene	21.1	ND	tr	0.24	tr	0.75	0.33	ND	ND	ND	0.31	ND
80	α-*trans*-Bergamotene	21.4	ND	ND	ND	ND	ND	ND	ND	ND	ND	0.81	ND
81	α-Guaiene	21.5	ND	ND	ND	ND	ND	ND	ND	ND	ND	2.69	ND
82	Aromadendrene	21.8	ND	ND	ND	ND	0.26	ND	ND	ND	ND	ND	ND
83	α-Humulene (α-Caryophyllene)	22.0	tr	0.13	0.50	0.21	2.42	0.20	0.16	ND	ND	1.87	3.72
84	allo-Aromadendrene	22.3	ND	ND	ND	ND	0.69	ND	ND	ND	ND	ND	ND
85	*cis*-Muurola-4(14),5-diene	22.5	ND	0.15	0.92	0.34	ND	ND	ND	ND	ND	0.63	ND
86	Germacrene D	23.2	0.91	1.08	2.32	0.77	14.56	3.64	ND	ND	ND	4.98	ND
87	Bicyclogermacrene	23.7	0.20	0.22	0.71	0.20	0.79	0.60	0.63	ND	ND	0.97	ND
88	α-Muurolene	24.0	ND	ND	ND	ND	0.30	ND	ND	ND	ND	ND	ND
89	α-Bulnesene (δ-Guaiene)	24.1	ND	ND	ND	ND	ND	ND	ND	ND	ND	4.90	ND
90	γ-Cadinene	24.6	ND	0.10	tr	ND	ND	ND	0.12	ND	ND	4.40	ND
91	β-Bisabolene	24.4	ND	ND	ND	ND	1.67	ND	ND	ND	0.11	ND	ND
92	γ-Cadinene	24.5	ND	ND	ND	ND	1.07	ND	ND	ND	ND	ND	ND
93	β-Bisabolene+γ-Cadinene	24.6	ND	ND	ND	ND	ND	0.34	ND	ND	ND	ND	ND
94	δ-Cadinene	24.9	ND	0.19	0.60	0.19	2.53	0.26	ND	0.11	ND	0.49	ND
95	Elemol	26.0	ND	ND	ND	ND	1.01	ND	ND	ND	ND	ND	ND
96	Germacrene D-4-ol	26.9	ND	ND	ND	ND	0.23	ND	ND	ND	ND	ND	ND
97	Spathulenol	27.0	ND	ND	ND	ND	ND	ND	ND	ND	ND	2.02	ND
98	Caryophyllene oxide	27.1	0.29	1.93	3.80	0.63	8.82	4.16	1.41	0.51	tr	ND	0.17
99	Viridiflorol	27.5	0.50	1.37	0.39	ND	ND	ND	ND	ND	ND	ND	3.46
100	Humulene epoxide II	28.1	ND	ND	ND	ND	0.76	ND	ND	ND	ND	ND	0.64
101	τ-Cadinol	29.4	ND	ND	ND	ND	0.60	ND	ND	ND	ND	10.93	ND
102	α-Cadinol	30.0	ND	0.36	0.51	0.22	1.50	ND	ND	ND	ND	0.62	ND
103	Bifomene	33.6	ND	ND	ND	ND	ND	ND	ND	ND	ND	ND	0.04
104	Manool	34.6	ND	ND	ND	ND	ND	ND	ND	ND	ND	ND	8.07
	Monoterpene hydrocarbons		6.06	5.37	4.04	8.11	5.88	35.23	42.89	36.89	37.86	0.87	12.21
	Sesquiterpene hydrocarbons		2.93	2.30	19.97	8.56	42.06	9.63	3.81	1.08	0.64	31.72	4.97
	Oxygenated monoterpenes		89.82	82.68	68.95	81.96	35.34	49.91	50.32	60.37	61.51	49.22	69.61
	Oxygenated sesquterpenes		0.80	3.66	4.70	0.85	12.93	4.16	1.41	0.51	0.00	13.57	4.27
	Other		0.20	2.01	1.19	0.52	0.63	0.50	0.00	0.34	0.00	0.00	8.11
	Total		99.80	96.02	98.84	100.00	96.84	99.43	98.43	99.19	100.00	95.38	99.17

^[a]^ RT—retention time (min); ND—not detected; tr—trace; MP1—Mentha piperita from Institute for Medicinal Plant Research “Dr Josif Pancic”; MP2—Mentha piperita from Adonis d.o.o.; MP3—Mentha piperita from Bilje Borča d.o.o.; MP4—Mentha piperita from agricultural holding, Banatska Topola; OB7—Ocimum basilicum from agricultural holding, Bačko Novo Selo; OM5—Origanum majorana from Geneza d.o.o.; OV1—Origanum vulgare from Institute for Medicinal Plant Research “Dr Josif Pancic”; OV3—Origanum vulgare from Bilje Borča d.o.o.; SO7—Salvia officinalis from agricultural holding, Bačko Novo Selo; SM3—Satureja montana from Bilje Borča d.o.o.; SH4—Satureja hortensis from agricultural holding, Banatska Topola; TV6—Thymus vulgaris from agricultural holding, Kulpin; MC—moisture content.

**Table 3:** GC-MS profile of EOs obtained from Asteraceae species.

No.	Component	RT ^[a]^	Relative Percentage (%)
			MC1
1	Camphor	9.9	0.14
2	Menthone	10.3	0.07
3	Isomenthone	10.7	0.05
4	Menthol	11.1	0.07
5	*trans*-Caryophyllene	20.7	0.11
6	α-Humulene (α-Caryophyllene)	22.0	tr
7	*trans*-β-Farnesene	22.4	4.84
8	Dihydro sesquicineole	22.8	0.39
9	Germacrene D	23.2	tr
10	β-Selinene	23.4	tr
11	δ-Cadinene	24.9	tr
12	Spathulenol	26.9	1.95
13	α-Bisabolol oxide B	29.8	19.93
14	α-Bisabolone oxide A	30.7	4.57
15	α-Bisabolol	30.8	6.17
16	Chamazulene	31.7	5.54
17	α-Bisabolol oxide A	31.9	30.64
18	*cis*-Spiroether	33.4	22.10
19	*trans*-Spiroether	33.6	0.44
	Monoterpene hydrocarbons		0.00
	Sesquiterpene hydrocarbons		10.49
	Oxygenated monoterpenes		0.34
	Oxygenated sesquiterpenes		63.26
	Other		22.92
	Total		97.01

^[a]^ RT—retention time (min); tr—trace; MC1—Matricaria chamomilla from Institute for Medicinal Plant Research “Dr. Josif Pancic”.

**Table 4:** GC-MS profile of EOs obtained from Apiaceae species.

No.	Component	RT ^[a]^	Relative Percentage (%)				
			PA1	FV7	CC1	AG7	PC1
1	α-Pinene	3.9	ND	2.33	ND	0.15	8.17
2	Camphene	4.2	ND	Tr	ND	ND	ND
3	Sabinene	4.8	ND	Tr	ND	ND	0.14
4	β-Pinene	4.8	ND	0.15	ND	ND	6.02
5	Myrcene	5.2	ND	0.55	tr	0.16	tr
6	α-Phellandrene	5.5	ND	0.73	ND	3.54	tr
7	*p*-Cymene	6.1	ND	0.08	ND	0.58	0.08
8	Limonene	6.2	ND	1.81	27.63	45.24	ND
9	β-Phellandrene	6.2	ND	ND	ND	ND	1.87
10	γ-Terpinene	7.1	ND	0.79	ND	ND	0.11
11	*cis*-Sabinene hydrate	7.4	ND	tr	ND	ND	ND
12	Fenchone	8.0	ND	15.48	ND	0.09	ND
13	Linalool	8.5	0.75	ND	ND	ND	ND
14	Camphor	9.9	ND	0.35	ND	ND	ND
15	Terpinen-4-ol	11.3	ND	tr	ND	ND	ND
16	Dill ether	11.5	ND	ND	ND	1.07	ND
17	α-Terpineol	11.9	ND	ND	0.11	ND	ND
18	Myrtenal	11.9	ND	ND	ND	ND	0.19
19	*cis*-Dihydrocarvone	12.0	ND	ND	0.48	0.73	ND
20	Estragole	12.1	1.20	3.81	ND	ND	ND
21	*trans*-Dihydrocarvone	12.3	ND	ND	0.14	1.77	ND
22	Isodihydrocarveol	12.8	ND	ND	0.12	0.11	ND
23	*trans*-Carveol	13.0	ND	ND	tr	0.08	ND
24	Neoisodihydrocarveol	13.3	ND	ND	0.29	0.26	ND
25	*cis*-Carveol	13.5	ND	ND	ND	0.12	ND
26	Carvone	13.8	0.14	ND	70.25	45.90	ND
27	*cis*-Anethole	14.3	0.10	0.06	ND	ND	ND
28	*p*-Anisaldehyde	14.5	tr	ND	ND	ND	ND
29	*trans*-Anethole	15.6	96.40	73.85	0.90	0.19	ND
30	*trans*-Caryophyllene	20.7	ND	ND	0.08	ND	ND
31	α-Himachalene	21.8	0.13	ND	ND	ND	ND
32	*trans*-β-Farnesene	22.4	ND	ND	ND	ND	0.02
33	γ-Himachalene	23.0	tr	ND	ND	ND	ND
34	Germacrene D	23.2	ND	tr	ND	tr	ND
35	Unknown	23.2	1.29	ND	ND	ND	ND
36	Dill apiole	29.3	ND	ND	ND	tr	ND
37	Myristicin	24.9	ND	ND	ND	ND	35.81
38	Elemicin	26.4	ND	ND	ND	ND	5.53
39	Carotol	27.6	ND	ND	ND	ND	0.26
40	6-Methoxyelemicin	27.9	ND	ND	ND	ND	17.44
41	Apiol	30.8	ND	ND	ND	ND	24.35
	Monoterpene hydrocarbons		0.00	6.45	27.63	49.68	16.39
	Sesquiterpene hydrocarbons		0.13	0.00	0.08	0.00	0.29
	Oxygenated monoterpenes		98.58	93.55	72.29	50.32	0.19
	Oxygenated sesquiterpenes		0.00	0.00	0.00	0.00	0.00
	Other		0.00	0.00	0.00	0.00	83.14
	Total		98.71	100.00	100.00	100.00	100.00

^[a]^ RT—retention time (min); ND—not detected; tr—trace; AG7—Anethum graveolens from agricultural holding, Bačko Novo Selo; CC1—Carum carvi from Institute for Medicinal Plant Research “Dr Josif Pancic”; FV7—Foeniculum vulgare from agricultural holding, Bačko Novo Selo; PC1—Petroselinum crispum from Medicinal Plant Research “Dr Josif Pancic”; PA1—Pimpinella anisum from Institute for Medicinal Plant Research “Dr Josif Pancic”.

**Table 5:** GC-MS profile of EOs obtained from Cupressaceae species.

No.	Component	RT ^[a]^	Relative Percentage (%)		
			JC2	JC3	JC1
1	α-Thujene	3.8	0.30	0.45	0.67
2	α-Pinene	3.9	33.06	44.73	29.57
3	Sabinene	4.7	13.12	11.83	19.64
4	β-Pinene	5.2	ND	ND	11.57
5	Myrcene	5.4	12.28	7.50	0.39
6	α-Terpinene	5.8	tr	tr	ND
7	*p*-Cymene	6.1	0.30	0.30	0.21
8	Limonene	6.2	4.80	4.45	4.92
9	γ-Terpinene	7.1	0.32	0.26	0.81
10	*cis*-Sabinene hydrate	7.4	tr	tr	tr
11	Terpinolene	8.0	0.62	0.62	1.02
12	Linalool	8.4	tr	tr	tr
13	Isoamyl isovalerate	8.5	tr	ND	ND
14	Borneol	10.8	tr	tr	tr
15	Terpinen-4-ol	11.2	1.30	1.23	1.42
16	α-Terpineol	11.9	0.28	tr	ND
17	Bornyl acetate	14.2	0.33	0.24	tr
18	1,5,5-Trimethyl-6-methylene-cyclohexene	17.4	0.17	0.13	0.43
19	α-Cubebene	17.9	0.99	0.57	0.63
20	α-Terpinyl acetate	18.1	0.17	tr	ND
21	α-Copaene	19.0	0.74	0.36	0.22
22	β-Elemene	19.7	2.22	1.68	1.75
23	Longifolene	20.0	0.30	0.10	0.13
24	*trans*-Caryophyllene	20.7	2.69	3.01	2.58
25	β-Gurjunene	21.1	0.40	0.21	0.27
26	γ-Elemene	21.3	3.36	5.03	3.78
27	α-Humulene (α-Caryophyllene)	22.0	2.19	2.25	2.17
28	*trans*-β-Farnesene	22.4	0.66	0.37	0.34
29	γ-Muurolene	23.1	0.19	0.23	0.30
30	Germacrene D	23.2	8.02	4.36	7.59
31	β-Selinene	23.4	0.33	0.46	0.25
32	Bicyclogermacrene	23.7	0.72	0.62	1.01
33	α-Muurolene	24.0	0.32	0.37	0.34
34	γ-Cadinene	24.5	0.39	0.45	0.36
35	δ-Cadinene	24.9	2.53	1.66	1.99
36	Germacrene D-4-ol	27.0	0.81	0.67	0.75
37	Caryophyllene oxide	27.1	1.30	0.73	0.70
38	τ-Cadinol	29.5	1.42	0.97	0.69
39	α-Cardinol	30.0	1.98	1.50	1.30
	Monoterpene hydrocarbons		64.96	70.27	69.24
	Sesquiterpene hydrocarbons		26.04	21.74	23.70
	Oxygenated monoterpenes		2.09	1.47	1.42
	Oxygenated sesquiterpenes		5.51	3.86	3.45
	Other		0.00	0.00	0.00
	Total		98.60	97.34	97.81

^[a]^ RT—retention time (min); ND—not detected; tr—trace; JC2—Juniperus comunis from Adonis d.o.o.; JC3—Juniperus comunis from Bilje Borča d.o.o.; JC1—Juniperus comunis from Institute for Medicinal Plant Research “Dr Josif Pancic”.

**Table 6:** DPPH and ABTS^+^ radical scavenging activity of EOs obtained from Apiaceae, Asteraceae, Cupressaceae and Lamiaceae species.

Family	Plant Name English	Plant Name Latin	Sample Name	DPPH Assay			ABTS Assay		
				c [mg/mL]	DPPH [µM TE/g]	Std	c [mg/mL]	ABTS [µM TE/g]	Std
Apiaceae	Dill	*Anethum graveolens*	AG7	10	2.86	0.3898	10	50.89	1.6416
	Caraway	*Carum carvi*	CC1	15	0.46	0.4836	10	27.61	0.3570
	Fennel	*Foeniculum vulgare*	FV7	10	1.37	0.3898	10	28.40	0.6554
	Parsley	*Petroselinum crispum*	PC1	20	1.52	0.9029	10	24.95	0.3825
	Anise	*Pimpinella anisum*	PA1	20	2.26	0.3683	10	40.34	0.7803
Asteraceae	Echinacea	*Ehinacea purpurea*	EP2	10	3.97	0.8931	1	343.89	1.1899
	Chamomile	*Matricaria chamomilla*	MC1	10	44.20	0.8202	1	469.23	3.6353
Cupressaceae	Juniper	*Juniperus comunis*	JC3	15	3.38	0.2455	1	284.79	0.6870
			JC1	15	2.96	0.8173	1	324.06	1.8176
			JC2	15	4.15	0.8729	1	315.33	3.5698
Lamiaceae	Peppermint	*Mentha x Piperita*	MP1	15	5.71	0.5893	10	57.79	2.0135
			MP2	15	8.46	0.1299	1	301.45	4.1789
			MP3	20	1.96	0.1328	1	296.69	2.9946
			MP4	15	5.82	1.4542	10	50.77	2.9252
	Basil	*Ocimum basilicum*	OB7	15	44.97	1.1948	1	757.98	1.1899
	Marjoram	*Origanum majorana*	OM5	15	11.35	1.5806	1	477.56	4.1789
	Oregano	*Origanum vulgare*	OV1	10	2.22	1.0700	1	556.88	5.1868
	Sage	*Salvia officinalis*	SO7	10	1.03	2.2135	10	40.02	0.1818
	Winter savory	*Satureja montana*	SM3	15	3.55	0.2140	1	398.23	3.6353
	Summer savory	*Satureja hortensis*	SH4	10	23.32	1.0312	1	757.58	0.6870
	Thyme	*Thymus vulgaris*	TV6	10	29.78	1.3522	1	757.19	0.6870

Values expressed are means ± S.D. of three parallel measurements. DPPH—2,2-diphenyl-1-picrylhydrazyl; ABTS—2,2′-azino-bis (3-ethylbenzothiazoline-6-sulphonic acid; TE—Trolox equivalents; Std—standard deviation; AG7—Anethum graveolens from agricultural holding, Bačko Novo Selo; CC1—Carum carvi from Institute for Medicinal Plant Research “Dr Josif Pancic”; FV7—Foeniculum vulgare from agricultural holding, Bačko Novo Selo; PC1—Petroselinum crispum from Medicinal Plant Research “Dr Josif Pancic”; PA1—Pimpinella anisum from Institute for Medicinal Plant Research “Dr Josif Pancic”; EP2—Ehinacea purpurea from Adonis d.o.o.; MC1—Matricaria chamomilla from Institute for Medicinal Plant Research “Dr Josif Pancic”; JC2—Juniperus comunis from Adonis d.o.o.; JC3—Juniperus comunis from Bilje Borča d.o.o.; JC1—Juniperus comunis from Institute for Medicinal Plant Research “Dr Josif Pancic”; MP1—Mentha piperita from Institute for Medicinal Plant Research “Dr Josif Pancic”; MP2—Mentha piperita from Adonis d.o.o.; MP3—Mentha piperita from Bilje Borča d.o.o.; MP4—Mentha piperita from agricultural holding, Banatska Topola; OB7—Ocimum basilicum from agricultural holding, Bačko Novo Selo; OM5—Origanum majorana from Geneza d.o.o.; OV1—Origanum vulgare from Institute for Medicinal Plant Research “Dr Josif Pancic”; SO7—Salvia officinalis from agricultural holding, Bačko Novo Selo; SM3—Satureja montana from Bilje Borča d.o.o.; SH4—Satureja hortensis from agricultural holding, Banatska Topola; TV6—Thymus vulgaris from agricultural holding, Kulpin.

**Table 7:** Contribution of corresponding variables (%) to the first three principal components.

Variable	F1 (35.33%)	F2 (20.62%)	F3 (18.28%)
EO yield	14.67	0.11	2.73
DPPH [µM TE/g]	19.69	13.82	0.02
ABTS [µM TE/g]	23.14	0.32	9.65
Monoterpene hydrocarbons	0.30	46.66	2.28
Sesquiterpene hydrocarbons	12.38	7.32	2.72
Oxygenated monoterpenes	12.98	24.85	14.80
Oxygenated sesquiterpenes	16.83	6.83	10.45
Other	0.00	0.09	57.35

## Data Availability

Not applicable.

## Supplementary Materials

The following supporting information can be downloaded at: https://www.mdpi.com/article/10.3390/plants12040745/s1, Table S1: Factor coordinates.
