# Supplementary material for: Essential Oils from Apiaceae, Asteraceae, Cupressaceae and Lamiaceae Families Grown in Serbia: Comparative Chemical Profiling with In Vitro Antioxidant Activity
Source: Plants (Basel). 2023 Feb 7;12(4):745. doi: 10.3390/plants12040745 (PMC9968228; doi:10.3390/plants12040745)
Supplement: Supplementary file 1 [file plants-12-00745-s001.zip › plants-2158883-supplementary.pdf]

SUPPLEMENTARY MATERIAL

Table S1. Factor coordinates.

	F1	F2	F3
**EO**	-0.644	0.043	0.200
DPPH	0.746	-0.477	0.016
ABTS	0.809	-0.073	-0.376
MoH	0.093	0.877	-0.183
SeH	0.592	0.347	-0.199
OxM	-0.606	-0.640	-0.465
OxS	0.690	-0.336	0.391
Other	0.004	0.037	0.916
AG7	-1.350	0.852	0.009
CC1	-1.441	-0.051	-0.215
FV7	-3.102	-0.863	0.285
PC1	-0.912	0.828	4.122
PA1	-1.900	-1.249	-0.272
MC1	3.865	-1.964	2.427
JC3	0.580	2.467	-0.118
JC1	0.563	2.502	-0.143
JC2	0.866	2.339	-0.170
MP1	-1.661	-0.989	-0.190
MP2	-0.371	-1.147	-0.508
MP3	-0.067	-0.435	-0.519
MP4	-1.170	-0.718	-0.273
OB7	2.854	-1.382	-0.743
OM5	0.409	0.348	-0.712
OV1	1.805	0.357	-0.648
SO7	-1.229	-0.417	0.294
SM3	0.291	0.433	-0.585
SH4	0.907	-0.368	-1.062
TV6	1.062	-0.542	-0.980

AG7 – *Anethum graveolens* from agricultural holding, Bačko Novo Selo; CC1 – *Carum carvi* from Institute from Medicinal Plant Research "Dr Josif Pancic"; FV7 – *Foeniculum vulgare* from agricultural holding, Bačko Novo Selo; PC1 – *Petroselinum crispum* from Medicinal Plant Research "Dr Josif Pancic"; PA1 – *Pimpinella anisum* from Institute for Medicinal Plant Research "Dr Josif Pancic"; MC1 – *Matricaria chamomilla* from Institute for Medicinal Plant Research "Dr Josif Pancic"; JC2 – *Juniperus comunis* from Adonis d.o.o.; JC3 – *Juniperus comunis* from Bilje Borča d.o.o.; JC1 – *Juniperus comunis* from Institute for Medicinal Plant Research "Dr Josif Pancic"; MP1 – *Mentha piperita* from Institute for Medicinal Plant Research "Dr Josif Pancic"; MP2 – *Mentha piperita* from Adonis d.o.o.; MP3 – *Mentha piperita* from Bilje Borča d.o.o.; MP4 – *Mentha piperita* from agricultural holding, Banatska Topola;

OB7 – *Ocimum basilicum* from agricultural holding, Bačko Novo Selo; OM5 – *Origanum majorana* from Geneza d.o.o.; OV1 – *Origanum vulgare* from Institute for Medicinal Plant Research "Dr Josif Pancic; SO7 – *Salvia officinalis* from agricultural holding, Bačko Novo Selo; SM3 – *Satureja montana* from Bilje Borča d.o.o.; SH4 – *Satureja hortensis* from agricultural holding, Banatska Topola; TV6 – *Thymus vulgaris* from agricultural holding, Kulpin;
F1-F2-F3 – factor coordinates
